# Supplementary material for: Potential and challenges for an integrated management of tuberculosis, diabetes mellitus, and hypertension: A scoping review protocol
Source: PLoS One. 2022 Jul 12;17(7):e0271323. doi: 10.1371/journal.pone.0271323 (PMC9275721; doi:10.1371/journal.pone.0271323)
Supplement: Attachment — Submitted filename: Rev2_Letter to Reviewer_Protocol_Widyaningsih, et al.docx [file pone.0271323.s002.docx]

**RESPONSES TO REVIEWERS**

| **Comments to the Author** | **Responses to reviewers’ comments** |
| --- | --- |
| Journal Requirements:  Please ensure that your manuscript meets PLOS ONE's style requirements, including those for file naming. The PLOS ONE style templates can be found at <https://journals.plos.org/plosone/s/file?id=wjVg/> PLOSOne_formatting_sample_main_body.pdf  <https://journals.plos.org/plosone/s/file?id=ba62/>  PLOSOne_formatting_sample_title_authors_affiliations.pdf | Thank you for your feedback. We recheck the formatting of the manuscript as well as the file name. |
| Please ensure that you refer to Figure 1 in your text as, if accepted, production will need this reference to link the reader to the figure. | Thank you, we have updated the reference to the figure, by changing from figure 2 to figure 1. The corresponding sentence has been changed as shown in Line 145-146.  “ Two authors will review the full text of selected studies according to Figure 1 independently.” |
| Please upload a copy of Figure 2, to which you refer in your text on page 8. If the figure is no longer to be included as part of the submission please remove all reference to it within the text. | Thank you, we have updated the reference to the figure, by changing from figure 2 to figure 1. There is only 1 figure in this manuscript. The corresponding sentence has been changed as shown in Line 144-145.  “ Two authors will review the full text of selected studies according to Figure 1 independently.” |
|  |  |
| **Reviewer #1:** | Thank you for the feedback and comments provided for this paper. We appreciate the time and efforts of reviewers and editor in providing feedback, that has |
| 1. In line 148 (Stage 5): The author should explain in more detail about collating, summarizing, and reporting results. | 1. Thank you, we revised line 158 (Stage 5) to explain about collating, summarizing, and reporting in more detail. |
| 2. In line 178 the parentheses have not closed. | 2. Thank you, we have made some changes and ensure the parentheses were closed. (Line 179-197)   “The results will be consulted to the experts and relevant stakeholders including regulators (health officers and national insurance officers), clinicians, and patient. This step is important to validate the findings, receive feedback and obtain additional insights into the findings.  Experts from India and Indonesia on TB, DM, and hypertension management will be invited for in-depth interviews. India and Indonesia contributes to more than one-third of TB burden in the world (4). Additionally, the two countries are also among the countries with highest number of Diabetes Mellitus and hypertension (29,30). The selection of the experts will consider their research background and/or program implementation experiences in TB, DM and hypertension management. After presentation of the finding, the discussion will be centered on whether the findings are applicable in the context of healthcare in India and Indonesia and whether there are additional information on potentials and barrier for integrated management of the diseases.”   3. |
| 3. In general, the discussion section is very short and concise, and it is better for the author to rewrite it in more detail. | 4. Thank you, we have elaborate the discussion section, and provide more information. (Line 227-250) |
| **Reviewer #2:**  **General Comments :** The introduction is well written and relevant citations are used. Overall, I think English needs improvement, authors use verb terms inconsistently. I would also suggest naming the sections of the protocol as per PLOS ONE requirements (https://journals.plos.org/plosone/s/what-we-publish/#loc-study-protocols) including the background, rationale, objectives, methodology, statistical plan, and organization of a research project. Content of the introduction section can be written under background and rationale; section, the identifying the research question, can be renamed as objective, all the rest can go under methodology and organization of the research project. I understand that a statistical plan is not relevant to your protocol. | Thank you, we revised few verb terms to make the explanation in introduction section more consistent.  Thank you for the suggestion, we have reviewed the guidelines and make necessary changes, with the following section:  Introduction  Material and Methods (all the steps are included in this section)  Discussions |
| **Abstract :**  Line 33: Please specify key search terms for TB, DM, and Hypertension. | Thank you, we revised this section to make the key terms clearer in line 35-36, as follow:  “key terms related to integrated management i.e., screening, diagnosis, treatment and care, of tuberculosis, DM, and hypertension” |
| Line 36: Please specify in which citation manager software the results will be exported. | Thank you, we use EndNote as a citation manager software. We add this information in line 42.  “We will export the search results to citation manager software (EndNote).” |
| **Introduction :**  Lines 65, 66 : Please mention the name of the author, as you do in the sentence before, otherwise it seems like that this finding is also from the same article (citation 16) and not from another article (citation 17). | Thank you, we add the author’s name of citation 17 in line 72-73. We have already checked articles 16 and 17 again, and it shows us that the findings we mentioned in lines 70-73 are from different articles.  “A previous study also showed an association between TB and hypertension. Chung et al. reported a higher prevalence of hypertension (38.7% in the tuberculosis group vs. 37.5% in the non-tuberculosis group) (16). A study by Mandieka et al, also showed that people with latent tuberculosis have a higher risk of developing hypertension (17).” |
| Lines 71-72 : Please provide a citation. | Thank you, the finding in line 71-72 is also from the same article of citation 21. We have provided the citation |
| Lines 74-75: Please mention: "… and DM and Cardiovascular events …", I understand that mortality rates were higher in both groups: Hypertension and DM and Cardiovascular events and DM vs Hypertension alone and Cardiovascular events alone | Thank you, we revised the sentence to make it more clear Line 84-86  “Compared to people who only had DM or hypertension, people with DM and hypertension comorbidities had higher rate of mortality (21) and are at increased risk for cardiovascular events (22). ” |
| Lines 76-78 : Not clear to me what do you mean. It is clear from previous citations (19-21) that DM is significantly associated with hypertension but citation 22 is interpreted so that TB can be an effect modifier among patients with DM. I believe that is not true. Could you please revise? | Thank you, we rephrase the sentence. What we meant, was that compared to people with only TB, people with TB-DM comorbidities had higher cardiovascular risk. We revise the sentence in line 85-90.  “Meanwhile, among TB patients, those who also have DM are at increased risk for cardiovascular diseases compared to those with only TB [24.5% vs. 5.5%]. Furthermore, TB patients with DM comorbidities are also more likely to use antihypertensive medication compared to patients with only TB diagnosis (16.9% vs. 3.2%) (23). |
| **Materials and Methods :**  Lines 98-99: Why do you include studies published between January 2005 and July 2021, and not, for instance, including December 2021? | Thanks for your comment. We include studies published between January 2005 and July 2021 because we have completed the research protocol before July 2021 so that the next stage can be carried out: data collection until July 2021 |
| Lines 99-100: Have you already conducted the scoping review in Aug-Dec 2021 as it is mentioned in the protocol? If so, please ignore my comments above. If not, please revise the timeline. | Yes, we already conducted the scoping review in Aug-Dec 2021 as it is mentioned in the protocol. |
| **Stage 1. Identifying the research question**  Lines 103, 104: I would suggest splitting the research question into two research questions:   a. What is the potential for integrated management of Tuberculosis, Diabetes Mellitus, or Hypertension ?” b. What are the challenges for integrated management of Tuberculosis, Diabetes Mellitus, or Hypertension ?” | Thanks for your comment. We revised the research questions as your suggestions (see line 113-117). |
| **Stage 2: Identifying relevant studies**  Line 118, Table 1: Could you please clarify whether the study population included patients only with TB or only with DM, or only with Hypertension? | Thanks for your comment. The study population are patients with 2 diseases (DM with hypertension or DM with TB or HT with TB) and patients with 3 diseases (DM, TB and hypertension). We have revised the sentence (in Table 1) to better clarify that we are interested in finding people with comorbidities of these diseases.  “Target population included people with comorbidities of tuberculosis and/or diabetes and/or hypertension, i.e., people with tuberculosis and hypertension or diabetes, people with hypertension and diabetes, or people with the three diseases.” |
| If so, how would you learn about integrating the management of TB with Hypertension, TB with DM, or DM and Hypertension in these groups? | Thanks for your comment. By looking at the symptoms experienced by the patient, the drugs that the patient gets are associated with existing treatment algorithms  We included this additional information in the concept (Table 1):  “Integrated tuberculosis, diabetes mellitus, hypertension management,(screening, diagnosis, treatment or care). These include screening, diagnosis, and treatment of people with tuberculosis and hypertension or diabetes, people with hypertension and diabetes, and people with the three diseases.” |
| Also, is it your interest to review integrated management of TB and NCDs or are you also interested in reviewing integrated care of NCDs only, i.e., DM and Hypertension in your protocol? | Thanks for your comment. We interested in exploring existing integrated management of comorbidities among three diseases. Hence, we will also include hypertension and diabetes mellitus in our search. We have included additional information in table 1. |
| The column of Context suggests specifying indicators for the program implementation process (input, process output, and outcome) and indicators at different levels of implementation (patient, institution, health system, region, district). | Thanks for your comment, which will clarify this protocol. We have specified the indicators of context in table 1, as follow:  “All types of indicators (input, process, output) of disease management, i.e. resources needed (human resources, funding, equipment, system), fidelity and effectiveness of existing integrated management implementation. “ |
| Would be good to see an annex with an exact list of inclusion and exclusion criteria. | Thanks for your comment. We embedded inclusion and exclusion criteria in this table, by categorizing this into the population, concept, context, and type of sources. However, we realize we haven’t stated the exclusion criteria the context. Hence, we provided additional information in Table 1 as follow:  “As the aim of this scoping review is to explore potential and challenges for integrated management of these diseases, we plan to include all studies explaining the context of the disease management, with no specific exclusion criteria for context. |
| **Stage 3: Study selection**  Line 139: Figure1. Flow chart for the study selection is missing | Thanks for your comment. We will include it on revision, as a separate tiff (image file) |
| **Stage 4: Charting the data**  Line 146 Table 4. Extraction Data Templates: Quantitative, Qualitative, Mixed Methods, etc. are study design examples and I suggest renaming the raw: Article type into study design or study type. Also, I suggest including research article, study protocols, Review, etc. in the separate raw named article type. | Thanks for your comment. We revised the table and changed the title to table 3 (We only have 3 tables in this manuscript). Extraction Data Template by moving the data description from “Article Type” to “Study Design” as suggested. For “article type”, we will include original research for the scoping review, as well as guidelines, and unpublished reports from grey literature |
| **Stage 5: collating, summarizing, and reporting results**  Line 151: specify what do you mean by study location, e.g., high income vs Low middle-income countries? | Thanks for your comment. What we mean by study location is a country that conducted the research, both in high income and low middle-income countries. We also have included this in the document:  “study location (countries where the research were conducted, in high and low middle-income countries)” |
| **Stage 6: conducting consultation**  Not sure what do you mean under the results will be consulted to the experts and relevant stakeholders. Do you mean focus group interviews with experts and relevant stakeholders? I understand that you plan to discuss the results and identify additional constructs and validate your findings from the article review? Please clarify how will you select experts and relevant stockholders? Will the discussion be structured facilitated as per the specially designed template? What questions do you plan to ask? How would you report the outcomes of the consultation? | Thanks for your feedback and comment. Yes, we plan to conduct a focus group discussion with expert and relevant stakeholder. For each group of stakeholders, we will identify key person who can provide adequate information on the topic of integrated TB-DM and hypertension management. Expert from India and Indonesia on TB, DM, and hypertension management will be invited for the FGD. As for the stakeholder consultation, we plan to invite relevant stakeholders, particularly from Indonesia to validate and provide feedback to our findings. The stakeholders include the Ministry of Health officials coordinating TB and NCD management, district health officials, and the officials from the national health insurance. A separate FGD with patients with TB and DM or hypertension comorbidities will be conducted. The patients FGD is conducted in a type A hospital in Central Java, Indonesia. This setting is chosen due to the high prevalence of TB, DM, and hypertension in this province.  The discussion will be structured and focus on the reliability and applicability of our findings in the context of India and Indonesia. The FGDs will be recorded, and verbatim transcript will be produced. Analyses of the theme and subtheme derived from the FGDs will be presented in the scoping review to provide more comprehensive information.  To clarify the steps in the text, we add the following information (Line 183-210):  This step is important to validate the findings, receive feedback and obtain additional insights into the findings.  Experts from India and Indonesia on TB, DM, and hypertension management will be invited for in-depth interviews. India and Indonesia contributes to more than one-third of TB burden in the world (4). Additionally, the two countries are also among the countries with highest number of Diabetes Mellitus and hypertension (30,31). The selection of the experts will consider their research background and/or program implementation experiences in TB, DM and hypertension management. After presentation of the finding, the discussion will be centered on whether the findings are applicable in the context of healthcare in India and Indonesia and whether there is additional information on potentials and barrier for integrated management of the diseases.  Meanwhile, the stakeholders consultations will be conducted as focus group discussions, in which the findings will be presented and discussed. As for the stakeholder consultation, we plan to invite relevant stakeholders, particularly from Indonesia to validate and provide feedback to our findings. For each group of stakeholders, key person who can provide adequate information on the topic of integrated TB-DM and hypertension management will be identified. The stakeholder include the Ministry of Health officials coordinating TB and NCD management, district health officials, and the officials from the national health insurance. A separate FGD with patients with TB and DM or hypertension comorbidities will be conducted. The patients FGD is conducted in several communities and hospitals in Central Java Province, Indonesia. This setting is chosen due to the high prevalence of TB, DM, and hypertension in this province. Central Java is also one of prioritized provinces for TB and NCD interventions in Indonesia. |
| **Discussion**  I suggest removing the discussion section. You can report about the limitations in your publication. | Thanks for your comment. We consider that this discussion section is an important part, hence we include this in our study protocol. However, with the brief discussion section, we think that we need to elaborate this to incorporate not only limitation and strength of the study. We include the following information in our discussion section (line 227-250):  “This scoping review will provide information on potential and challenges for integrated management of tuberculosis, DM, and hypertension, with different stage and level of integration. A systematic review on integrating TB and NCDs care in Low-middle income countries (LMICs) highlighted the benefit of integrated disease management in improvement of health service delivery, particularly, if comprehensive management from screening, referral, and treatment is conducted (31). However, in this scoping review, we will focus on TB, DM and hypertension integration. Our scoping review will strengthen the arguments on the implementation of TB-DM integrated management. It is because increased case detection through bi-directional screening lead to more effective management of the diseases (32). The integration reduced treatment loss to follow up for both TB-DM, with increasing treatment success among TB patients (33). However, synthesis of the integration among three diseases, i.e., TB, DM and hypertension is still lacking. |
