# Supplementary material for: Potential and challenges for an integrated management of tuberculosis, diabetes mellitus, and hypertension: A scoping review protocol
Source: PLoS One. 2022 Jul 12;17(7):e0271323. doi: 10.1371/journal.pone.0271323 (PMC9275721; doi:10.1371/journal.pone.0271323)
Supplement: Attachment — Submitted filename: Response to Reviewer_rev2.docx [file pone.0271323.s003.docx]

| Comments to the Author  1. Does the manuscript provide a valid rationale for the proposed study, with clearly identified and justified research questions?  The research question outlined is expected to address a valid academic problem or topic and contribute to the base of knowledge in the field.  Reviewer #2: Yes | Response  Thanks for your complement. |
| --- | --- |
| 2. Is the protocol technically sound and planned in a manner that will lead to a meaningful outcome and allow testing the stated hypotheses?  The manuscript should describe the methods in sufficient detail to prevent undisclosed flexibility in the experimental procedure or analysis pipeline, including sufficient outcome-neutral conditions (e.g. necessary controls, absence of floor or ceiling effects) to test the proposed hypotheses and a statistical power analysis where applicable. As there may be aspects of the methodology and analysis which can only be refined once the work is undertaken, authors should outline potential assumptions and explicitly describe what aspects of the proposed analyses, if any, are exploratory.  Reviewer #2: Partly | Thanks for your assessment. We have revised the manuscript as the reviewers’ suggestion. |
| 3. Is the methodology feasible and described in sufficient detail to allow the work to be replicable?  Descriptions of methods and materials in the protocol should be reported in sufficient detail for another researcher to reproduce all experiments and analyses. The protocol should describe the appropriate controls, sample size calculations, and replication needed to ensure that the data are robust and reproducible.  Reviewer #2: Yes | Thank you. |
| 4. Have the authors described where all data underlying the findings will be made available when the study is complete?  The PLOS Data policy requires authors to make all data underlying the findings described in their manuscript fully available without restriction, with rare exception, at the time of publication. The data should be provided as part of the manuscript or its supporting information, or deposited to a public repository. For example, in addition to summary statistics, the data points behind means, medians and variance measures should be available. If there are restrictions on publicly sharing data—e.g. participant privacy or use of data from a third party—those must be specified.  Reviewer #2: No | Thank you. Our manuscript is a scoping review protocol, hence, the data have not available yet. |
| 5. Is the manuscript presented in an intelligible fashion and written in standard English? 🡪  PLOS ONE does not copyedit accepted manuscripts, so the language in submitted articles must be clear, correct, and unambiguous. Any typographical or grammatical errors should be corrected at revision, so please note any specific errors here.  Reviewer #2: No | Thank you. We have done English editing throughout the manuscript. |
| 6. Review Comments to the Author  Please use the space provided to explain your answers to the questions above and, if applicable, provide comments about issues authors must address before this protocol can be accepted for publication. You may also include additional comments for the author, including concerns about research or publication ethics. |  |
| **Reviewer #2: General comments:** |  |
| 1. To use abbreviations, you’ll first want to spell out the name or phrase, followed by the abbreviation in parentheses. Then, in any subsequent use of that name or phrase, only use the abbreviation.  Examples. In each case, you write out the full name and then introduce the abbreviation in parentheses: the word "tuberculosis (TB)" becomes the “TB” and the phrase “non-communicable disease (NCD)" becomes “NCD”. | Thank you. We have revised the sentences as you suggested. Please refer to line 28-37 and:  “This scoping review discusses the possibilities and problems of integration in managing TB and NCDs, with a particular emphasis on diabetic mellitus (DM) and hypertension screening and control”  ” We will use key terms related to integrated management, i.e., screening, diagnosis, treatment, and care, of TB, DM, and hypertension in PubMed, Scopus Database, and ScienceDirect for research published from January 2005 to July 2021.” |
| 2. Please make sure that the grammar and style used are for academic writing, please ask for assistance if needed. | Thank you. We have revised the grammar and style. |
| 3. Some of the sentences are in the past tense and some of them are in the future tense. My understanding is that since this is the study protocol, all sentences must be in the future tense, but I see that study according to this protocol has already been conducted. If that is so, I do not know what to recommend. Dear Editor, please provide guidance on that | Thank you. We have revised the grammar to the future tense. Please refer to line 34-37:  ” We will use key terms related to integrated management, i.e., screening, diagnosis, treatment, and care, of TB, DM, and hypertension in PubMed, Scopus Database, and ScienceDirect for research published from January 2005 to July 2021” |
| 4. Study Protocols must also include the status and timeline of the study, including whether participant recruitment or data collection has begun where and when the data will be made available. See our Data Availability policy for more. | Thank you for your feedback. We have updated the information in the methods section, as follow (line 103-104):  “The scoping review will be conducted from August 2021 to June 2022, starting with literature search, database creation and extraction.” |
| 5. I would encourage you to remove the discussion section and use parts of it in the introduction and in the Materials and Methods. (X) | Thanks for your comment. We receive comment from the other reviewer to elaborate the discussion section. In addition to that, several previous protocol papers published in this journal include discussion section. Hence, we opted to include discussion in our study protocol. |
| 6. Line 117, Table 1. Inclusion and exclusion criteria. The table is not clear enough. I have several questions/comments:  1. Did you include people with TB and hypertension?  2. Do you have age limitation for your study population?  3. I am not sure how this group (DM and hypertension) of the population addresses your research question; I understand your research questions are about the management of TB and NCDs (i.e., hypertension and DM); thus your population must have TB and at least one of the NCDs.  4. Do you exclude studies about people with TB only? Yes  5. Do you exclude studies about all pregnant women, or the only the ones with gestational diabetes and/or preeclampsia/eclampsia? Yes | Thank you for your feedback. To response to your questions. Please find our response per each question, as follow:  1. Yes, and we further clarify this in the text.  “Through this scoping review, we aim to present examples and potentials and challenges of integrating the management of TB, DM, and hypertension in any combinations i.e., TB and DM, TB and hypertension, and DM and hypertension.” (line 110-114)    2. Thank you. We only limit the search to adult population, due to the relatively low prevalence of NCD in children and the differences in children NCDs compared to adults. We include this information on table 1, population. (line 124, Table 1, column 1)  3. Thank you for your thoughtful feedback. As our focus in on multimorbidity of chronic diseases, with the main diseases of interest are those with high burden, hence, in this paper we aim to explore the possibility of the integration of the three diseases, in any combination. Hence, we will be able to identify programs that are already running which address the comorbidities of the three diseases and assess/evaluate the possibilities of integration of all the three diseases. For example, if there are programs addressing screening of DM and hypertension in the community, we’d like to explore if there might be a potential to include TB into these programs.  4. Yes, we will exclude studies only focusing on one disease. We elaborate this information on the table, as follow:  “Excluding papers with a focus on single disease screening, diagnosis and treatment” (line 124, Table 1 column 2)  5. We will exclude all studies on pregnant women, as the specific condition with existing maternal healthcare program, might require a more thorough review beyond the scope of this study. Please refer to line 124, Table 1, column 1 |
| Additional comments:  Abstract  Line 34-36, "This review will also 35 consider grey literature, including international disease management guidelines on tuberculosis, DM, and hypertension." Could you please specify what kind of gray literature other than clinical practice guidelines are you considering and what you mean by "international"? Do you mean WHO guidelines?  Lines 40-41, delete "will be synthesized"  Line 41, I suggest wording, the results will be used instead of could be used | Thank you. We have added information about grey literature  “To obtain a comprehensive view on integrated management of Tuberculosis, hypertension, and diabetes, we will include original papers, grey literature including unpublished literature and international disease management guidelines on TB, DM, and hypertension from WHO or other health professional organization” (line 99-103)  Based on your suggestion, we also have revised the sentences on line 44-46:  “We will synthesize all selected studies qualitatively and the results will be discussed with the experts. The results will be used to guide the development of a guideline for integrated TB, DM, and hypertension management.” |
| Introduction  Line 63, please clarify what "previous study" you mean, as it is mentioned at the beginning of a new paragraph it is unclear what study you mean  Lines 95 -96, Could you please specify what kind of gray literature other than clinical practice guidelines are you considering and what you mean by "international"? Do you mean WHO guidelines? Do you consider the guidelines part of gray literature? I read from the text that you plan to review gray literature and international guidelines. | Thank for your comments. The previous studies include Chung *et al,* Mandieka *et al.,* and Seegert *et al.* Please refer to line 66-73:  “Several study also showed an association between TB and hypertension. Chung *et al*. reported a higher prevalence of hypertension (38.7% in the TB group vs 37.5% in the non-TB group) (16). A study by Mandieka et al*.*, also showed that people with latent TB have a higher risk of developing hypertension (17). In a meta-analysis by Seegert et al., the prevalence of hypertension in TB patients ranges from 0.7% to 38.3%; however, there is no significant difference in hypertension cases between TB and non-TB patients in most case-control studies (18)” |
| Stage 1: identifying the research question  Line 105, "… the management of any of hypertension, diabetes, and tuberculosis in any..", delete of any.  Line 112. correct spelling of "seatch" 🡪 search  Lines 114-115, "Hence, providing a review of literature for the past 15 years. Next, the title and abstract will be analyzed. " - something is missing, unclear to me, please clarify. | We have revised the sentence on line 119-122:  “The literature search will be conducted in three databases: PubMed, Scopus Database, and ScienceDirect from January 2005 to July 2021. Hence, providing a review of literature for the past 15 years. Next, the title and abstract will be analyzed for relevancy with the research aims” |
